# Three new species of *Dacryobolus* (*Polyporales*, *Basidiomycota*) from southern China

**DOI:** 10.3897/mycokeys.133.188064

**Published:** 2026-06-02

**Authors:** Qi-Zhi Zhu, Jing Ye, Man-Rong Huang, Shuang-Hui He, Yuan Yuan

**Affiliations:** 1 School of Ecology and Nature Conservation, Beijing Forestry University, Beijing 100083, China School of Ecology and Nature Conservation, Beijing Forestry University Beijing China; 2 Department of Life Sciences, Natural History Museum of China, Beijing 100050, China Department of Life Sciences, Natural History Museum of China Beijing China

**Keywords:** Brown rot, corticioid fungi, phylogeny, taxonomy, wood-decaying fungi

## Abstract

*Dacryobolus (Dacryobolaceae)* is a brown-rot corticioid genus distributed mainly in temperate and subtropical regions. In this study, specimens of *Dacryobolus* collected on *Pinus yunnanensis* from Yunnan and Sichuan Provinces, southwestern China, were studied by morphological and molecular methods. As a result, three new lineages within the clade of the genus were recognized based on the concatenated ITS–nrLSU phylogenetic tree. Accordingly, three new species, *Dacryobolus caesius*, *D. odontoides*, and *D. yunnanensis*, are described and illustrated for these new lineages. Morphologically, *Dacryobolus caesius* and *D. odontoides* have odontoid hymenophores and lack cystidia, whereas *D. yunnanensis* has smooth hymenophores and possesses thick-walled skeletocystidia. The taxonomy and phylogeny of *Dacryobolus* are discussed, and a key to all known species of the genus worldwide is provided. This study enriches knowledge about the species diversity of *Dacryobolus* and brown-rot corticioid fungi in China.

## Introduction

*Dacryobolus*, typified by *Hydnum sudans* Alb. & Schwein., is a small genus in *Polyporales* (Binder et al. 2013; Ortiz-Santana et al. 2013; Justo et al. 2017). Species of the genus usually produce resupinate, effused basidiomes with smooth to slightly tuberculate or distinctly odontoid hymenophores, a monomitic or dimitic hyphal system, nodose-septate generative hyphae, the absence or presence of one to two kinds of cystidia (thick-walled skeletocystidia and thin-walled hymenial cystidia), and narrowly cylindrical to allantoid, colorless, thin-walled, smooth basidiospores negative in Melzer’s reagent and cotton blue (Eriksson and Ryvarden 1975; Bernicchia and Gorjón 2010; Gorjón 2020). Ecologically, all species cause brown rot on both angiosperm and gymnosperm branches or trunks. Phylogenetically, *Dacryobolus* is placed in the *Dacryobolaceae* of the “*Antrodia*” clade in *Polyporales*, but the relationship with other genera, such as *Auriporia* Ryvarden and *Sarcoporia* P. Karst., needs further study (Ortiz-Santana et al. 2013; Justo et al. 2017). At present, seven species are recognized in the genus, and four of them have been recorded in China (Yuan et al. 2016; Xu et al. 2018). Nevertheless, because of the complex climatic conditions and diverse forest types in southern China, the species diversity of *Dacryobolus* in this region remains insufficiently explored.

*Pinus yunnanensis* Franch. is an endemic species mainly distributed in Yunnan, Sichuan, and Guizhou Provinces and Guangxi Autonomous Region, southwestern China, and plays an important role in the ecological stability and economic development of these areas (Fu et al. 1999). Meanwhile, *Pinus yunnanensis* is an ideal host for both ectomycorrhizal and wood-decaying fungi, and many species associated with it have been found and described with the aid of molecular evidence (Yu et al. 2007; Li et al. 2025; Xiang et al. 2025; Zhou et al. 2025). Recently, several specimens of *Dacryobolus* were collected on rotten branches and trunks of *Pinus yunnanensis* from Yunnan and Sichuan Provinces during intensive surveys of wood-decaying fungi on this host. Phylogenetic studies showed that they did not belong to any of the known species but represented three new lineages. Therefore, combined with morphological evidence, three new species of this genus are described and illustrated as follows. This study contributes to knowledge about the species diversity of corticioid fungi on *Pinus yunnanensis* in China.

## Materials and methods

### Specimen collection

Specimens were dried with a portable dryer, labeled, and then stored in a freezer at –40 °C for 2 weeks to kill the insects and their eggs before proceeding with morphological and molecular studies. Voucher specimens are deposited at the herbarium of Beijing Forestry University, Beijing, China (**BJFC)**.

### Morphological studies

Thin, freehand sections were made from dried basidiomes and mounted in 2% (w/v) aqueous potassium hydroxide (KOH) and 1% (w/v) aqueous phloxine. Amyloidity and dextrinoidity of microstructures were checked in Melzer’s reagent (IKI). Cyanophily of microstructures was observed in 1% (w/v) cotton blue in 60% (w/v) lactic acid (CB). Microscopic examinations were carried out with a Nikon Eclipse 80i microscope (Nikon Corporation, Japan) at magnifications up to 1000×. Drawings were made with the aid of a drawing tube. The following abbreviations are used: IKI– = neither amyloid nor dextrinoid, CB– = acyanophilous, *L* = mean spore length, *W* = mean spore width, *Q* = *L*/*W* ratio, *n* (*a*/*b*) = number of spores (*a*) measured from the number of specimens (*b*).

### DNA extraction and sequencing

A CTAB plant genomic DNA extraction kit, DN14 (Aidlab Biotechnologies Co., Ltd., Beijing, China), was used to extract total genomic DNA from dried specimens, which was then amplified by polymerase chain reaction (PCR) according to the manufacturer’s instructions. The ITS1-5.8S-ITS2 region was amplified with the primer pair ITS5/ITS4 (White et al. 1990), whereas the D1-D2 region of the nuclear ribosomal LSU was amplified with the primer pair LR0R/LR7 (https://sites.duke.edu/vilgalyslab/rdna_primers_for_fungi/). DNA sequencing was performed at Beijing Genomics Institute, and newly generated sequences were deposited in GenBank (https://www.ncbi.nlm.nih.gov/). BioEdit v.7.0.5.3 (Hall 1999) was used to review the chromatograms.

### Phylogenetic analyses

The dataset of concatenated ITS1-5.8S-ITS2-nrLSU sequences of *Dacryobolus* and related taxa in *Dacryobolaceae* was analyzed. *Antrodia subserpens* B.K. Cui & Yuan Y. Chen and *A. kmetii* Vlasák were selected as the outgroup (Xu et al. 2018). The concatenated sequences were aligned using MAFFT v.74 (http://mafft.cbrc.jp/alignment/server/, Katoh et al. 2017) with the G-INS-I iterative refinement algorithm and optimized manually in BioEdit v.7.0.5.3. Maximum parsimony (MP), Bayesian inference (BI), and maximum likelihood (ML) analyses were carried out using PAUP* v.4.0b10 (Swofford 2002), MrBayes 3.2.6 (Ronquist et al. 2012), and RAxML v.8.2.10 (Stamatakis 2014), respectively. In ML analyses, statistical support values were obtained using rapid bootstrapping with 1000 replicates, with default settings used for other parameters. For BI, four Markov chains were run for 2,000,000 generations for the dataset until the split deviation frequency value was lower than 0.01. Trees were sampled every 100^th^ generation. The first quarter of the trees, which represented the burn-in phase of the analyses, was discarded, and the remaining trees were used to calculate posterior probabilities (BPP) in the majority-rule consensus tree.

## Results

### Phylogenetic analyses

**Figure 1.**
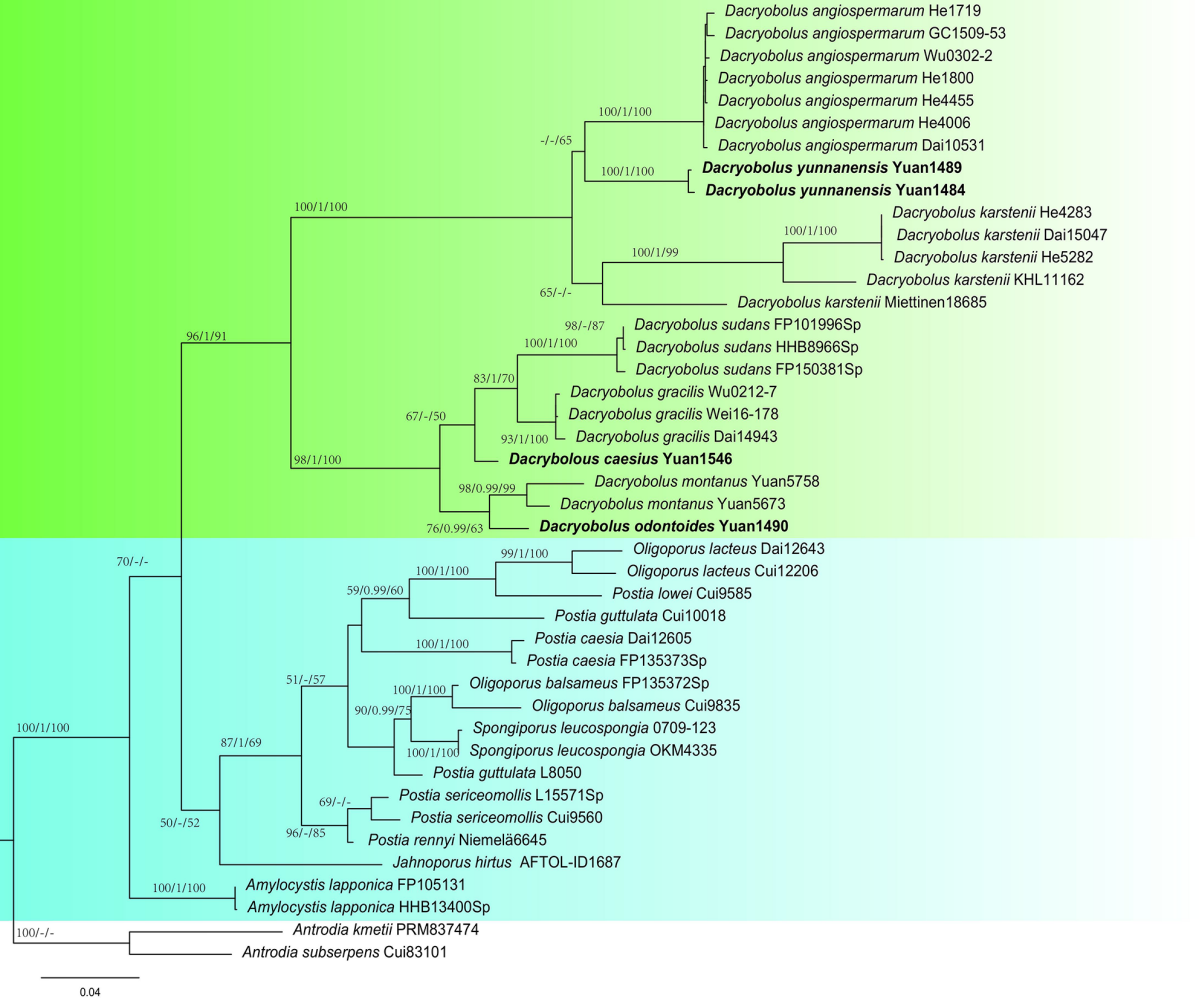
Phylogenetic tree obtained from maximum likelihood analyses of the combined ITS and nrLSU sequences of *Dacryobolaceae*. Branches are labeled with parsimony bootstrap values (≥ 50%, first), Bayesian posterior probabilities (≥ 0.95, second), and likelihood bootstrap values (≥ 50%, third). New species are set in bold.

The combined ITS–nrLSU dataset contained 43 samples representing 18 ingroup taxa and the outgroup. Four ITS and two nrLSU sequences were newly generated in this study (Table 1). The aligned dataset contained 1983 characters, of which 1407 were constant, 101 were parsimony-uninformative, and 475 were parsimony-informative. MP analysis yielded 24 equally parsimonious trees. The MP and BI analyses resulted in tree topologies almost identical to those of the ML analyses. Only the ML tree is shown in Fig. 1, with parsimony bootstrap values (≥ 50%, first), Bayesian posterior probabilities (≥ 0.95, second), and likelihood bootstrap values (≥ 50%, third) labeled along the branches.

**Table 1.** Species and sequences used in the phylogenetic analyses. New species are set in bold, with type specimens indicated with an asterisk (*).

Taxa	Voucher	Locality	ITS	nLSU
*Amylocystis lapponica*	FP 105131	USA	KY948805	KY948879
	HHB 13400 Sp	USA	KC585237	KC585059
*Antrodia kmetii*	PRM 837474	Croatia	KC886710	KT995153
*A. subserpens*	Cui 8310	China	KP715310	KP715326
*Dacryobolus angiospermarum*	He 1719	China	MH048962	MH048975
	He 1800	China	MH048963	MH048976
	He 4006	China	MH048965	MH048978
	He 4455*	China	MH048964	MH048977
	Dai 105313	China	MH048966	MH048979
	Wu 0302-2	China: Taiwan	MH048967	MH048980
	GC 1509-53	China: Taiwan	MH048968	MH048981
***D. caesius***	Yuan 1546*	**China**	**PX929864**	**–**
*D. gracilis*	Dai 14943	China	MH048972	MH048985
	Wu 0212-7	China: Taiwan	MH048973	MH048986
	Wei 16-178	China: Taiwan	MH048974	MH048987
*D. karstenii*	He 4283	China	MH048970	MH048983
	He 5282	China	MH048969	MH048982
	Dai 15047	China	MH048971	MH048984
	KHL 11162	Sweden	EU118624	EU118624
	Miettinen 18685	USA	KY948743	KY948900
*D. montanus*	Yuan 5673*	China	KC344410	KC344411
	Yuan 5758	China	KC344412	KC344413
***D. odontoides***	Yuan 1490*	**China**	**PX929863**	**–**
*D. sudans*	FP 101996 Sp	USA	KC585332	KC585157
	FP 150381 Sp	USA	KC585333	KC585158
	HHB 8966 Sp	USA	KC585334	KC585159
***D. yunnanensis***	**Yuan 1484**	**China**	**PX929861**	**PX929865**
	Yuan 1489*	**China**	**PX929862**	**PX929866**
*Jahnoporus hirtus*	AFTOL-ID 1687	USA	DQ911605	DQ911606
*Oligoporus balsameus*	Cui 9835	China	KX900916	KX900986
	FP 135372 Sp	United Kingdom	KC585358	KC585187
*Oligoporus lacteus*	Cui 12206	China	KR605820	KR605763
	Dai 12643	China	KF699121	KJ684981
*Postia caesia*	Dai 12605	Finland	KX900883	KX900953
	FP 135373 Sp	United Kingdom	KC585375	KC585205
*P. guttulata*	Cui 10018	China	KF727432	KJ684978
	L 8050	USA	KC585360	KC585189
*P. lowei*	Cui 9585	China	KX900898	KX900968
*P. rennyi*	Niemelä 6645	Finland	KC595929	KC595929
*P. sericeomollis*	Cui 9560	China	KX900919	KX900989
	L 15571 Sp	USA	KC585363	KC585192
*Spongiporus leucospongia*	0709-123	USA	KX900918	KX900988
	OKM 4335	USA	KC585395	KC585228

In the phylogenetic tree, *Dacryobolus* formed a strongly supported monophyletic clade (96/1/91), and two subclades, *D. sudans* (Alb. & Schwein.) Fr. (generic type) and *D. karstenii* (Bres.) Oberw. ex Parmasto, corresponding to taxa with odontoid and smooth hymenophores, can be recognized. The three new species, *Dacryobolus caesius*, *D. odontoides*, and *D. yunnanensis*, formed distinct lineages that could not be assigned to known species.

### Taxonomy

#### Dacryobolus caesius

Taxon classificationFungiPolyporalesDacryobolaceae

Q.Z. Zhu, Yuan Yuan & S.H. He sp. nov.

DF6CBC3B-AF39-5836-8DF2-B34499F8FCE5

862346

Fig. 2

##### Type.

China • Sichuan Province, Yanyuan County, Lugu Lake Scenic Area, on fallen branches of *Pinus yunnanensis*, 21 Jul 2024, Yuan Yuan, Yuan 1546 (BJFC 053959, holotype).

**Figure 2.**
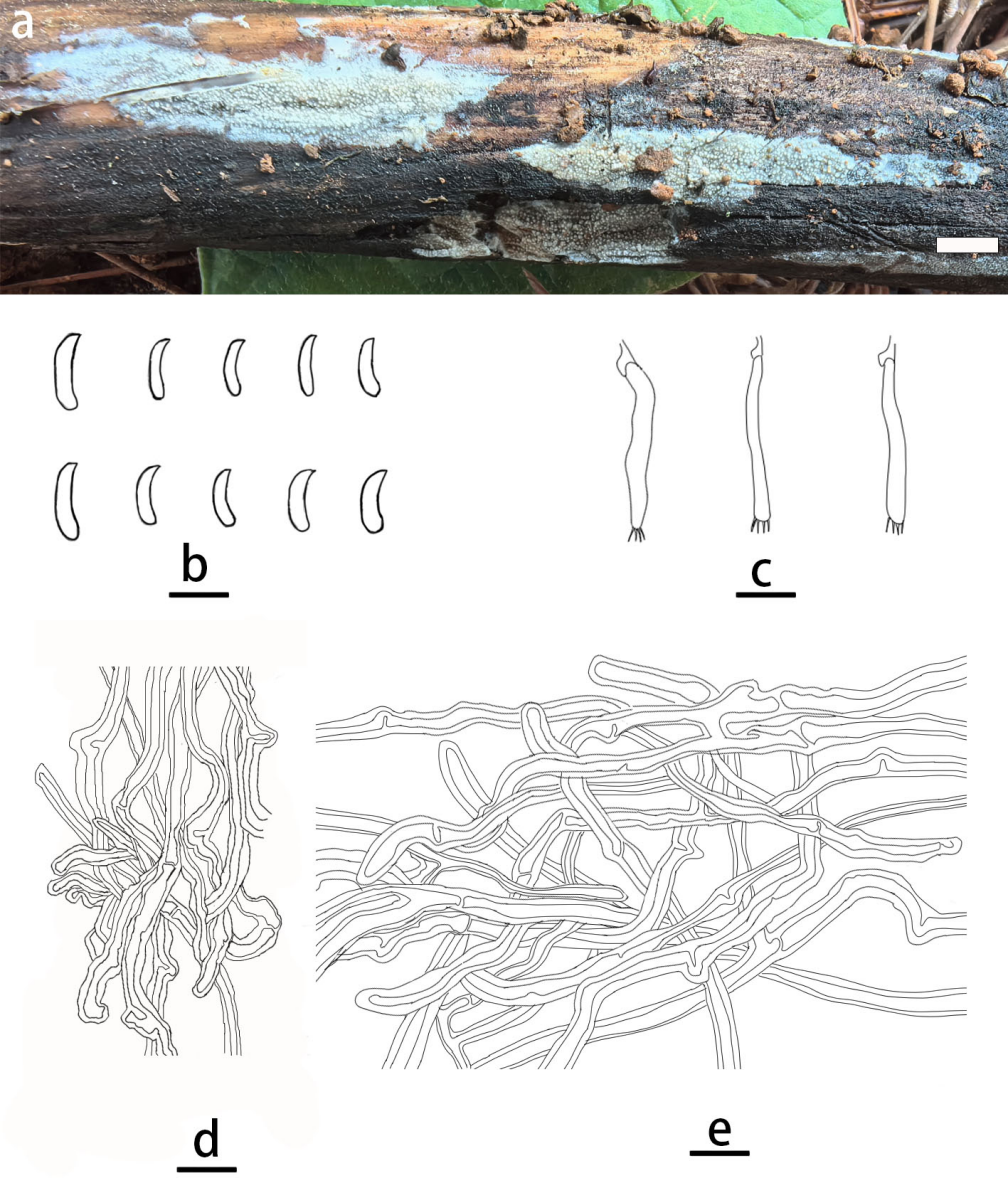
*Dacryobolus caesius* (from Yuan 1546, the holotype). **a**. Basidiomes; **b**. Basidiospores; **c**. Basidia; **d**. Hyphae from aculei; **e**. Hyphae from subiculum. Scale bars: 1 cm (**a**); 5 µm (**b**); 10 µm (**c–e**).

##### Etymology.

Refers to the bluish-gray basidiomes when fresh.

##### Fruiting body.

Basidiomes annual, resupinate, effused, closely adnate to the substrate, slightly ceraceous when fresh, becoming coriaceous to horny after dry, forming irregular patches or narrow strips extending along the longitudinal axis of the wood, confluent up to 7 cm long, 1 cm wide. Hymenophore odontoid, grayish white, pale bluish white to light gray when fresh, slightly glossy when moist, becoming grayish white to pale grayish yellow when dry, unchanged in KOH, not cracked; aculei conical to cylindrical, unbranched, solitary, rather uniform under the naked eyes, 2–3 per mm, up to 0.5 mm long, 0.4 mm wide at base; margin thinning out, determined, closely adnate, indistinct, concolorous with hymenophore surface.

##### Microscopic structures.

Subiculum thin and tightly adnate to the substrate. Hyphal system monomitic; generative hyphae with clamp connections and secondary simple septa, IKI–, CB–; subicular hyphae colorless, slightly thick- to thick-walled, smooth, moderately branched, interwoven, 3.5–5 μm in diam., occasionally with short tubular lateral branches perpendicular to the main hyphae; tramal hyphae colorless, thin- to slightly thick-walled, smooth, occasionally branched, 1.5–4.5 μm in diam., more or less parallel arranged. Cystidia absent. Basidia narrowly clavate, colorless, thin-walled, smooth, with a basal clamp connection and four sterigmata, 27–35 × 3–3.5 μm; basidioles similar in shape but slightly smaller. Basidiospores allantoid, colorless, thin-walled, smooth, IKI–, CB–, 5.5–7 × 1–1.5 μm; *L* = 6 μm, *W* = 1.14 μm, *Q* = 5.26 (*n* = 30/1).

##### Notes.

*Dacryobolus caesius* is characterized by resupinate, odontoid, bluish-gray basidiomes with short, evenly distributed aculei, a monomitic hyphal system with clamped generative hyphae, the absence of cystidia, relatively long, narrow, allantoid basidiospores, and a habitat on *Pinus yunnanensis*. In the phylogenetic tree, *D. caesius* was nested within the *D. sudans* subclade, which included three known species with odontoid hymenophores: *D. sudans*, *D. gracilis* H.S. Yuan, and *D. montanus* X.Z. Wan & H.S. Yuan (Fig. 1). Morphologically, *D. sudans* and *D. gracilis* differ from *D. caesius* by the presence of cystidia, whereas *D. montanus* differs in having longer aculei (up to 1.5 mm) and growing on angiosperm trees (Yuan et al. 2016).

#### Dacryobolus odontoides

Taxon classificationFungiPolyporalesDacryobolaceae

Q.Z. Zhu, Yuan Yuan & S.H. He sp. nov.

F7A57448-F408-576E-8670-F161262B22BC

862347

Fig. 3

##### Type.

China • Yunnan Province, Lijiang County, Xiangshan Park, on decayed branches of *Pinus yunnanensis*, 20 Jul 2024, Yuan Yuan, Yuan 1490 (BJFC 053903, holotype).

**Figure 3.**
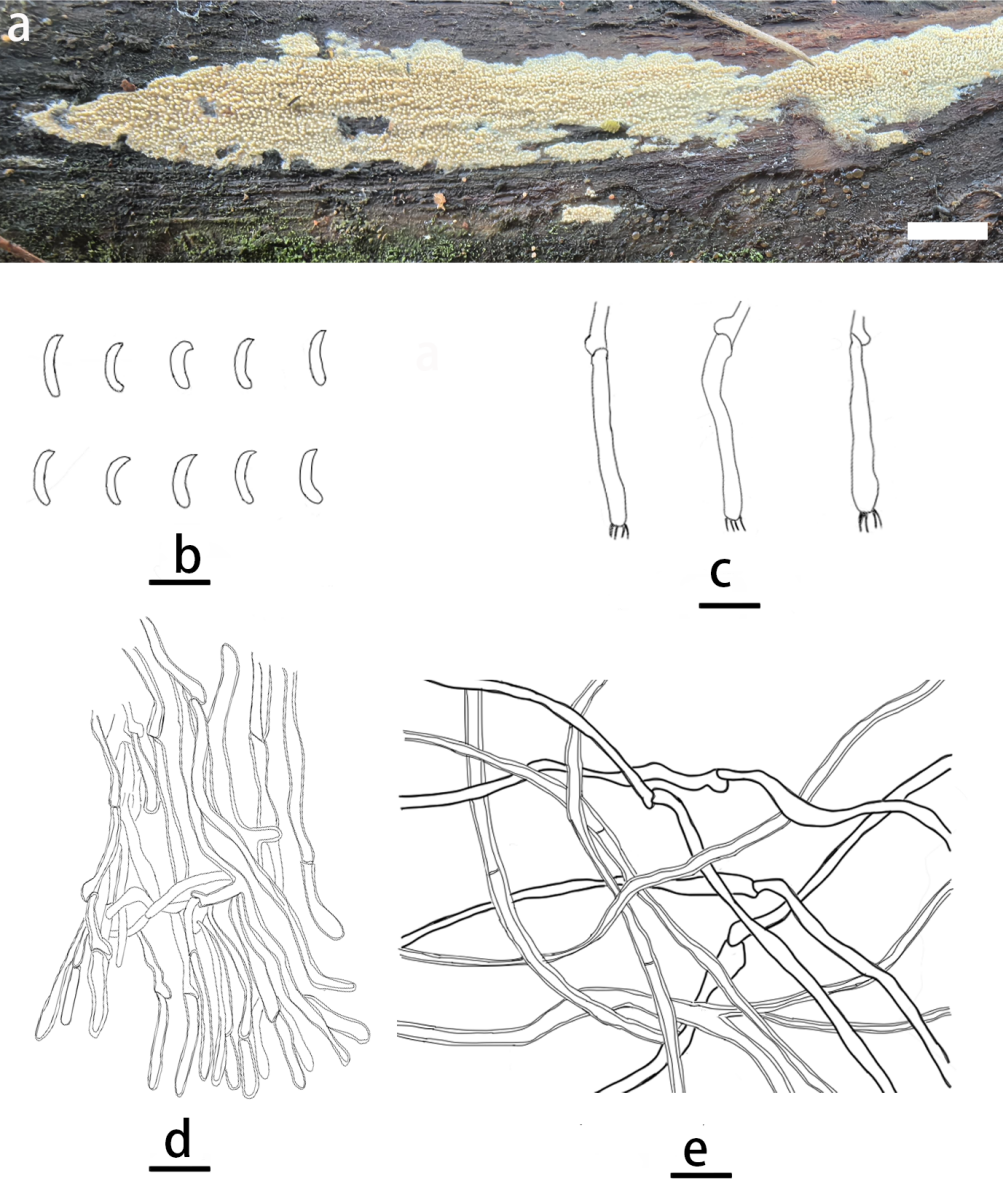
*Dacryobolus odontoides* (from Yuan 1490, the holotype). **a**. Basidiomes; **b**. Basidiospores; **c**. Basidia; **d**. Hyphae from aculei; **e**. Hyphae from subiculum. Scale bars: 1 cm (**a**); 5 µm (**b**); 10 µm (**c–e**).

##### Etymology.

Refers to the distinctly odontoid hymenophore.

##### Fruiting body.

Basidiomes annual, resupinate, effused, tightly adnate to the substrate, slightly ceraceous when fresh, becoming coriaceous to horny after dry, forming irregular patches often extending along wood fibers; individual patches several centimeters long and a few millimeters to 1–2 cm wide, usually confluent. Hymenophore odontoid, locally with uneven protuberances, granules more conspicuous in mature areas; pale yellow to light beige when fresh, slightly glossy when moist, becoming pale yellowish white to grayish yellow when dry, unchanged in KOH, not cracked; aculei conical to cylindrical, unbranched, 2–3 per mm, 0.3–0.4 mm wide at the base, up to 1 mm long; margin thinning out, determined, closely adnate, indistinct, concolorous with hymenophore surface.

##### Microscopic structures.

Subiculum extremely thin and hardly distinguishable. Hyphal system, monomitic; generative hyphae with clamp connections and secondary simple septa, IKI–, CB–; subicular hyphae colorless, slightly thick- to thick-walled, smooth, moderately branched, interwoven, 2–4 μm in diam., occasionally with short tubular lateral branches perpendicular to the main hyphae; tramal hyphae colorless, thin- to slightly thick-walled, smooth, occasionally branched, 1.4–2.2 μm in diam., more or less parallel arranged. Cystidia absent. Basidia narrowly clavate, colorless, thin-walled, smooth, with a basal clamp connection and four sterigmata, 18–20 × 2–3 μm; basidioles similar in shape but slightly smaller. Basidiospores allantoid, colorless, thin-walled, smooth, IKI–, CB–, 4.5–5.5 × 1–1.5 μm; *L* = 5.2 μm, *W* = 1.1 μm, *Q* = 4.7 (*n* = 30/1).

##### Notes.

*Dacryobolus odontoides* is distinguished by its odontoid hymenophore with conical to cylindrical aculei, a monomitic hyphal system with clamped generative hyphae, the absence of cystidia, relatively small, allantoid basidiospores, and a habitat on *Pinus yunnanensis*. Phylogenetically, *D. odontoides* is closely related to *D. montanus* within the *D. sudans* subclade. Morphologically, *D. montanus* differs from *D. odontoides* by having slightly longer aculei (up to 1.5 mm) and growing on angiosperm trees (Yuan et al. 2016). *Dacryobolus caesius*, described above, is similar to *D. odontoides* in sharing the odontoid hymenophore, the absence of cystidia, and a habitat on *Pinus yunnanensis* but differs in having bluish-gray basidiomes and slightly longer basidiospores.

#### Dacryobolus yunnanensis

Taxon classificationFungiPolyporalesDacryobolaceae

Q.Z. Zhu, Yuan Yuan & S.H. He sp. nov.

07FAC079-48F2-58C2-A40E-F82C243E4AED

862348

Fig. 4

##### Type.

China • Yunnan Province, Lijiang County, Xiangshan Park, on decayed branches of *Pinus yunnanensis*, 20 Jul 2024, Yuan Yuan, Yuan 1489 (BJFC 053902, holotype).

**Figure 4.**
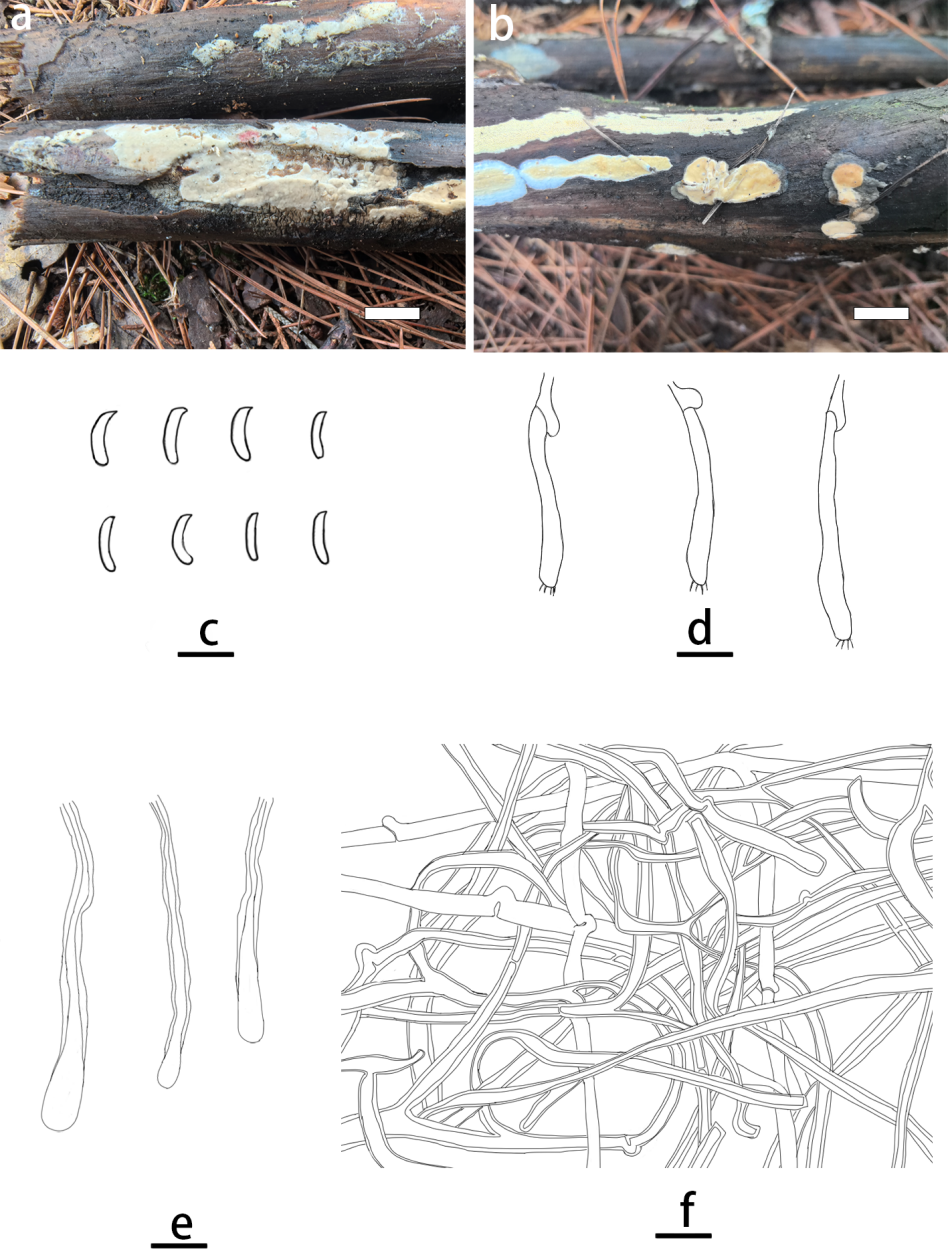
*Dacryobolus yunnanensis* (**a**, **c–f**. from Yuan 1489, the holotype; **b**. from Yuan 1484). **a, b**. Basidiomes; **c**. Basidiospores; **d**. Basidia; **e** .Skeletocystidia; **f**. Hyphae from subiculum. Scale bars: 1 cm (**a, b**); 5 µm (**c**); 10 µm (**e, f**).

##### Etymology.

Refers to the type locality in Yunnan Province, southwestern China.

##### Fruiting body.

Basidiomes annual, resupinate, effused, tightly adnate to the substrate, ceraceous to membranous when fresh, becoming coriaceous to soft corky after dry, at first as small irregular patches, later confluent up to 10 cm long, 3 cm wide, 1 mm thick in section. Hymenophore smooth to slightly verrucose, cream to pale beige when fresh and moist, becoming pale yellowish white to grayish white upon drying, unchanged in KOH, not cracked; margin thinning out, determined, closely adnate, distinct and white when juvenile, becoming indistinct and concolorous with hymenophore surface when mature.

##### Microscopic structures.

Hyphal system dimitic. Subiculum distinct; skeletal hyphae dominant, colorless, thick- to distinctly thick-walled, smooth, straight, unbranched, aseptate, swelling in KOH, 3–8 μm in diam., generative hyphae scattered, colorless, thin-walled, smooth, frequently branched and septate, with clamp connections, 2–4 μm in diam. Subhymenium thickening, composed of more or less vertically arranged hyphae and embedded cystidioid elements; hyphae slightly thick-walled, colorless, densely interwoven, slightly agglutinated, 2–3 μm in diam. Skeletocystidia cylindrical, colorless, thick-walled to subsolid, smooth, originating from the subiculum, usually with a long hyphal stalk, up to 110 μm long, 6 μm wide; projecting up to 50 μm beyond the hymenial surface; apical wall thin, gradually thickening toward the base; swelling in KOH. Basidia narrowly cylindrical, colorless, thin-walled, smooth, with four sterigmata and a basal clamp connection, 30–40 × 3–3.5 μm; basidioles similar in shape to basidia but slightly smaller. Basidiospores narrowly cylindrical to allantoid, colorless, thin-walled, smooth, IKI–, CB–, 5–6 × 1–1.5 μm; *L* = 5.3 μm, *W* = 1.2 μm, *Q* = 4.4 (*n* = 60/2).

##### Additional specimens examined.

China • Yunnan Province, Lijiang County, Xiangshan Park, on decayed branches of *Pinus yunnanensis*, 20 Jul 2024, Yuan Yuan, Yuan 1484 (BJFC 053897, paratype).

##### Notes.

*Dacryobolus yunnanensis* is characterized by resupinate, widely effused basidiomes with a smooth hymenophore, a dimitic hyphal system, the presence of skeletocystidia, and a habitat on *Pinus yunnanensis*. Phylogenetically, *D. yunnanensis* formed a distinct lineage within the *D. karstenii* subclade and was closely related to *D. angiospermarum* and *D. karstenii*. Morphologically, the three species are similar to each other in sharing smooth hymenophores, a dimitic hyphal system, and the presence of skeletocystidia; however, *D. angiospermarum* has so far been found on angiosperm trees in subtropical areas of Asia, and *D. karstenii* differs by having two types of cystidia (Bernicchia and Gorjón 2010; Xu et al. 2018). *Dacryobolus phalloides* Manjón, Hjortstam & G. Moreno also resembles *D. yunnanensis* by having smooth hymenophores and growing on *Pinus* wood but differs in having capitate skeletocystidia and slightly larger basidiospores (7–8 × 1.5–2 μm, Manjón et al. 1984).

## Discussion

Although *Dacryobolus* has been treated as a morphologically well-circumscribed genus (Bernicchia and Gorjón 2010), the monophyly of the genus is challenged as more species are subjected to phylogenetic analyses. Two distinct subclades, represented by the generic type *D. sudans* and the other common species *D. karstenii*, respectively, are recognized in the phylogenetic tree (Fig. 1). Meanwhile, there are different morphological patterns between the two groups. Species of the *D. sudans* group have odontoid basidiomes with a monomitic hyphal system and lack thick-walled skeletocystidia, whereas the *D. karstenii* group is characterized by smooth to slightly tuberculate hymenophores, a dimitic hyphal system, and the presence of skeletocystidia. Nevertheless, at present, all species are retained under a single genus to avoid too many small generic names.

Previous studies suggested that *D. sudans*, reported mainly from coniferous substrates in Europe and North America, may represent a species complex because of its wide morphological variation and compatibility patterns (Hallenberg 1985, 1991). Meanwhile, samples of *D. karstenii* collected from different areas formed distinct lineages in the phylogenetic trees (Xu et al. 2018; Fig. 1). Based on both morphological characteristics and phylogenetic analyses, the present study reveals three unknown species of *Dacryobolus* collected exclusively from southwestern China on *Pinus yunnanensis*. The results suggest a possible pattern of regional endemism and host specificity of *Dacryobolus* species. Meanwhile, this study expands the known diversity of the genus in China and highlights the importance of integrating morphological and molecular data in delimiting species of corticioid fungi. To date, 10 species of *Dacryobolus* are recognized worldwide, and six have been described from China.

### A key to the known species of *Dacryobolus* worldwide

**Table:** 

1	Hyphal system dimitic	**2**
–	Hyphal system monomitic	**4**
2	On angiosperm wood	***D. angiospermarum***
–	On gymnosperm wood	**3**
3	Cystidia of two kinds	***D. karstenii***
–	Cystidia of one kind	***D. yunnanensis***
4	Basidiocarps perennial; basidiospores suballantoid or ellipsoid	***D. costratus***
–	Basidiocarps annual; basidiospores subcylindrical to allantoid	**5**
5	Hymenophore smooth; basidiospores 7–8 × 1.5–2 µm	***D. phalloides***
–	Hymenophore odontoid with granular to cylindrical aculei; basidiospores < 7 µm long	**6**
6	Cystidia present	**7**
–	Cystidia absent	**8**
7	Aculei up to 0.5 mm long; cystidia of two kinds; basidiospores 5.5–7 × 1.4–1.9 µm	***D. sudans***
–	Aculei up to 1.5 mm long; cystidia of one kind; basidiospores 4.8–5.3 × 1–1.2 µm	***D. gracilis***
8	On angiosperm wood	***D. montanus***
–	On gymnosperm wood	**9**
9	Basidiomes bluish-gray; basidiospores 5.5–7 μm long	***D. caesius***
–	Basidiomes cream; basidiospores 4.5–5.5 μm long	***D. odontoides***

## Supplementary Material

XML Treatment for Dacryobolus caesius

XML Treatment for Dacryobolus odontoides

XML Treatment for Dacryobolus yunnanensis

## Additional information

### Artificial Intelligence (AI) use

The authors accept full responsibility for the content of the manuscript, including the disclosure of any use of AI.

No AI tools were used in the preparation of this manuscript.

### Author ORCIDs

Q.-Z. Zhu https://orcid.org/0009-0002-5072-5969

S.-H. He https://orcid.org/0000-0003-4702-3034

Y. Yuan https://orcid.org/0000-0001-6674-9848

### Data availability

All of the data that support the findings of this study are available in the main text.
